# Research on Simultaneous Measurement of Magnetic Field and Temperature Based on Petaloid Photonic Crystal Fiber Sensor

**DOI:** 10.3390/s23187940

**Published:** 2023-09-16

**Authors:** Lili Yan, Qichao Wang, Bin Yin, Shiying Xiao, Haisu Li, Muguang Wang, Xingyu Liu, Songhua Wu

**Affiliations:** 1 Faculty of Information Science and Engineering, Ocean University of China, Qingdao 266100, China; 2 Laoshan Laboratory, Qingdao 266237, China; 3 Institute of Lightwave Technology, Beijing Jiaotong University, Beijing 100044, China

**Keywords:** optical fiber magnetic field sensor, high-birefringence photonic crystal fiber, optical fiber interferometer, dual-parameter measurement

## Abstract

In this paper, we propose and design a magnetic field and temperature sensor using a novel petaloid photonic crystal fiber filled with magnetic fluid. The PCF achieves a high birefringence of more than 1.43 × 10^−2^ at the wavelength of 1550 nm via the design of material parameters, air hole shape and the distribution of the photonic crystal fiber. Further, in order to significantly improve the sensitivity of the sensor, the magnetic-fluid-sensitive material is injected into the pores of the designed photonic crystal fiber. Finally, the sensor adopts a Mach–Zehnder interferometer structure combined with the ultra-high birefringence of the proposed petaloid photonic crystal fiber. Magnetic field and temperature can be simultaneously measured via observing the spectral response of the x-polarization state and y-polarization state. As indicated via simulation analysis, the sensor can realize sensitivities to magnetic fields and temperatures at −1.943 nm/mT and 0.0686 nm/°C in the x-polarization state and −1.421 nm/mT and 0.0914 nm/°C in the y-polarization state. The sensor can realize the measurement of multiple parameters including temperature and magnetic intensity and has the advantage of high sensitivity.

## 1. Introduction

Magnetic field sensors are becoming more and more crucial in industrial and research applications, such as biomedical detection [1], space geophysics [2], controlled fusion in aerospace science [3], etc. Commonly used instruments for magnetic field detection are fluxgate magnetic field sensors [4], magnetic transistors, magneto-resistive (amorphous and giant magneto-resistor) sensors [5], Hall effect sensors [6] and magnetic diodes [2]. However, many of the sensors still suffer from low sensitivity, low stability and high power consumption. Considering inherent sensitivity to ambient magnetic field sources of magnetic field sensors, high measurement noise is another important disadvantage that cannot be ignored. Among the many disturbances in magnetic field detection, temperature disturbance is not negligible [7]. To meet the demands of high-precision magnetic field detection and overcome the limitations of traditional magnetic field sensors, there is an urgent need to design a highly sensitive sensor with simultaneous temperature and magnetic field detection.

The use of optical fiber sensors as an emerging technique is currently being carried out in magnetic field detection. Their advantages include high interference resistance, high sensitivity, and small size. Magnetic field sensors made by combining magneto-optical materials with optical fibers are the focus of this research. Among them, magneto-optical materials can directly and indirectly interact with optical parameters. Magnetofluid is a commonly used magneto-optical material with birefringence [8], the Faraday effect [9], field-dependent transmission [10], a tunable refractive index [11], and other magneto-optical properties. Using these properties, magnetofluid-based optical fiber devices have been realized.

An optical fiber magnetorheological magnetic field sensor mainly uses photonic crystal fiber (PCF) injected with magnetic fluid. PCF is an excellent-performing fiber with flexible aperture design and has the advantages of high birefringence [12,13], large mode field area [14], low confinement loss and high sensitivity [15,16]. Optical fiber magnetofluid magnetic field sensors can change the refractive index distribution of PCF by the injected filler [17]. Therefore, by using the flexible design characteristics of PCF and the magneto-optical capabilities of magneto-fluid, we can obtain a high-sensitivity, interference-resistant optical fiber magnetic field sensor [18]. This kind of sensor is currently receiving extensive attention and has provided preliminary results [19,20,21,22].

G. Wang et al. [23] developed a two-core PCF magnetic field sensor that is highly sensitive and has a short length of only 2000 µm. According to the testing results, the sensor can achieve a maximum sensitivity of −442.7 pm/Oe in the magnetic field between 30–520 Oe. J. Yin et al. [13] designed a compact, all-fiber vector magnetometer that can detect the direction and intensity of a magnetic field. The sensor showed a maximum sensitivity of transmission loss demodulation and wavelength demodulation of 1.79 dB/mT and 114.5 pm/mT, respectively. W. Zhang et al. [24] studied a magnetic field sensor whose maximum magnetic field sensitivity is 584 pm/Oe in the x-polarization mode and 700 pm/Oe in the y-polarization mode. The sensor achieves high sensitivity mainly by injecting magnetic fluid into the photonic crystal fiber. Since magnetic fluids are particularly susceptible to temperature changes in addition to magnetic fields, current optical fiber magnetofluid sensor designs require simultaneous temperature measurement.

Many researchers have put a lot of effort in the use of magnetofluid fiber optic dual parameter sensors to measure both temperatures and magnetic fields simultaneously. X. Mo et al. [25] created a D-shaped PCF-based SPR sensor in order to realize dual-parameter measurement at the same time. Its sensitivity to magnetic fields is 0.14274 nm/Oe and its sensitivity to temperature is −0.229 nm/°C. Y. Ying et al. [26] developed and studied a D-shaped PCF-based sensor which uses surface plasmon resonance and directional resonance coupling techniques to measure temperature and magnetic field simultaneously. It has a temperature sensitivity of roughly −1.25 nm/°C and a magnetic field sensitivity of roughly 0.21 nm/Oe. H. Liu et al. [27] also developed a sensor for simultaneous temperature and magnetic field measurements. The sensor is mainly realized by injecting temperature-sensitive materials into the dual-core PCF structure and has a temperature sensitivity of −0.37 nm/°C and a magnetic field sensitivity of 0.44 nm/mT. D. Wang et al. [28] used a PCF with regular circular pores to create a magnetic field and temperature sensor. The temperature sensitivity of the sensor is 7.6 nm/°C and the magnetic field sensitivity of the sensor is 0.75 nm/mT.

In this paper, we present a magnetic field sensor based on a petaloid PCF for dual-parameter temperature and magnetic field measurement. Firstly, we designed a petaloid PCF, which adopts a circular aperture design and a high-refractive-index bismuthate material substrate to better bind the energy in the fiber core to reduce energy loss. By adjusting the spacing and size of the air holes, the optimal PCF structure is obtained and achieves a high birefringence coefficient of 1.43 × 10^−2^ at 1550 nm. Such a structural design can well increase the birefringence coefficient, improve the resolution of the spectrum and facilitate the multi-parameter measurement of magnetic field and temperature. Then, we added magnetic fluid into air holes of the petaloid PCF. Magnetic fluid material is highly sensitive to temperature and magnetic field, which greatly improves the sensitivity of the magnetic field sensor to magnetic field and temperature. Finally, we use a Mach–Zehnder (MZI) system, which is able to measure temperature and magnetic field simultaneously via x- and y-polarized interference spectroscopy. By varying the length of the PCF and the length of the single-mode fiber (SMF), we came up with the optimal structure of the system which can effectively improve the sensitivity of magnetic field and temperature detection. Through research and analysis, the highest sensitivity of the sensor reaches −1.943 nm/mT and 0.0686 nm/°C for magnetic field and temperature in the x-polarized state, and −1.421 nm/mT and 0.0914 nm/°C in the y-polarized state. The proposed dual-parametric sensor can be well used for magnetic field detection and can overcome the interference of ambient temperature. At the same time, the sensor has a small size and simple structure, which can be well used in practice. Moreover, the proposed sensor has high sensitivity, meaning it has potential applications in geomagnetism detection, and biomedical and medical testing.

## 2. Materials and Methods

### 2.1. Magnetic Fluid

Magnetic fluid is an excellent photosensitive material which is rather sensitive to the magnetic field in its surroundings. It consists of a liquid carrier, a surfactant and magnetic nanoparticles with diameters within the nanometer range. It has stable colloidal properties. Magnetic nanoparticles include substances such as Fe, Ni, Co, etc. Liquid carrier substances include water, organic solvents, oil, etc. Oleic acid is usually used as a surfactant to prevent agglomeration. When magnetic nanoparticles are subjected to external magnetic field changes, in the direction of the magnetic field, an internal motion of nanoparticles is generated. As the magnetic field intensity keeps increasing, the particles will be arranged into flux chains. However, if all particles are aggregated into magnetic chains, they reach saturation and the refractive index will not change. The Langevin function [29] can be used to demonstrate that the temperature and magnetic field affect the refractive index (n_MF_) of a magnetic fluid:

(1) $$n_{MF}(H,T)=(n_{s}-n_{0})[\coth(\alpha \frac{H-H_{c,n}}{T})-\frac{T}{\alpha (H-H_{c,n})}]+n_{0},H>H_{c,n}$$

where H_c,n_ is the crucial magnetic field intensity if the magnetic fluid begins to change, $n_{s}$ is the saturated refractive index of magnetic nanoparticles, T is the temperature of the environment where the magnetic fluid is present, $n_{0}$ is the initial refractive index of the magnetic fluid and H is the external magnetic field intensity. The formula reveals that H and T are the two elements that have great impacts on the refractive index of magnetic fluid. From this point of view, the sensor made of magnetic fluid can realize the needs for magnetic field and temperature detection.

### 2.2. The Proposed Model of Petaloid PCF

**Figure 1:**
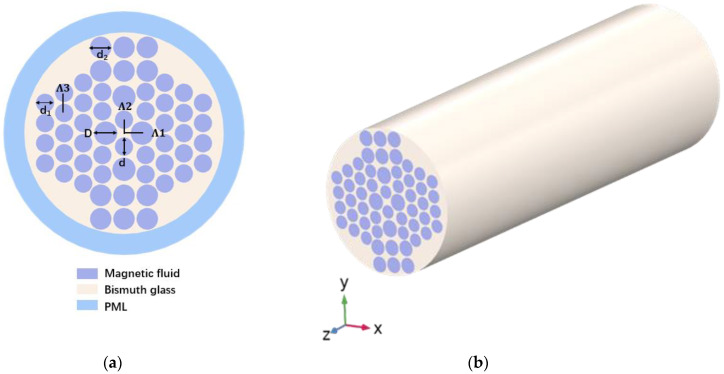
(**a**) Cross-sectional diagram and (**b**) 3D stereogram of the presented PCF.

The design of the PCF uses the method of finite element analysis [30]. Figure 1 shows the petaloid PCF cross-section model and 3D model. The structure of the PCF includes multi-layer air holes filled with magnetic fluid, and it utilizes the perfectly matched layer (PML) and the scattering boundary condition (SBC) of the outermost layer of the PCF to absorb the radiant energy from the surface. PML is a two-dimensional boundary that absorbs excess electromagnetic waves, and the refractive index can be set to the refractive index of the cladding. The SBC is able to transmit the normal light waves inside the model to be the normal light waves that are transmitted inside the geometric model. The cladding material of the PCF sensor is a composite bismuthate material [31], which has an ultra-high refractive index so that light can be well confined in the core. At the same time, the air holes inside near the center are designed to be larger, which is convenient for filling the magnetic fluid. They all involve the adoption of a circular hole design, which will be easier for future fabrication. Its *x*-axis and *y*-axis adopt an asymmetric structure, which effectively improves the birefringence characteristics. The diameters of the two largest holes on the left and right side of the fiber center are D, and the diameters of the circular holes above and below the fiber center are d. The diameters of the remaining holes are d_1_ = 1.1 µm and d_2_ = 1.3 µm. Λ_1_ and Λ_2_ are the distances between the fiber center and the circular hole center. Λ_3_ is the *y*-direction distance of the outer triangular unit cell around the center air hole. It is set to be 2.4 µm. The size of the four dimensions D, d, Λ_1_ and Λ_2_ will be discussed later.

The refractive index of the magnetic fluid will vary as the outside magnetic field intensity and temperature change. Then, it will affect the effective mode refractive index of the PCF and lead to the change in birefringence, B, of the PCF:

(2) $$B=|n_{eff}{}^{x}-n_{eff}{}^{y}|$$

where n_eff_^x^ is the effective mode refractive index of x polarization, and n_eff_^y^ is the effective mode refractive index of y polarization.

**Figure 2:**
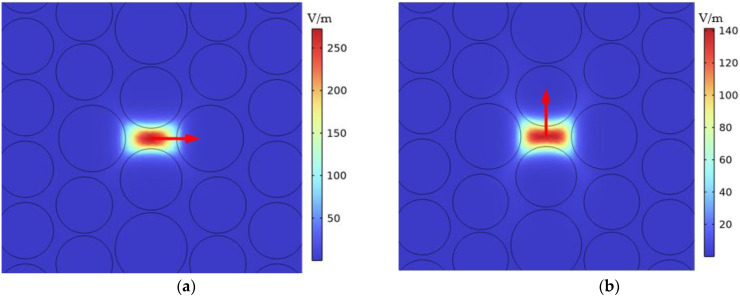
Mode field distribution of the petaloid PCF at a wavelength of 1550 nm with the (**a**) x-polarization state and (**b**) y-polarization state.

As shown in Figure 2, the *x* direction and the *y* direction can stably form the polarization state and achieve a stable birefringence effect. The red arrow represents the direction of the electric field.

### 2.3. Optical Path Structure Design of Sensor

**Figure 3:**
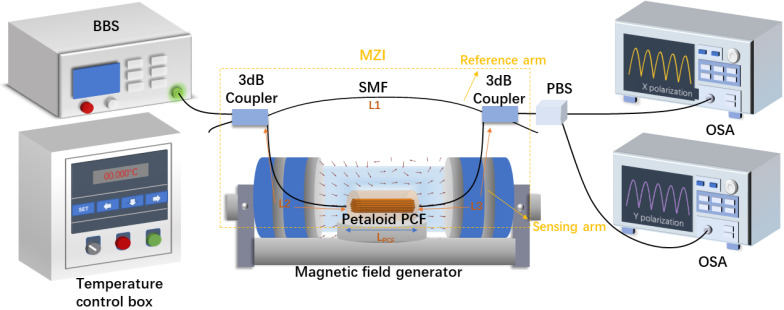
The test schematic of the proposed MZI-sensing system based on the petaloid PCF.

Figure 3 illustrates the optical transmission path and system structure of the optical fiber magnetic fluid sensor. The light comes from a broadband light source (BBS) and is split into two beams by the first 3 dB coupler; one beam only passes through the SMF as a reference arm and the other beam passes through the designed PCF as a sensing arm. Next, the two beams converge at the second 3 dB coupler and are split into two paths by a polarization beam splitter (PBS) into two optical spectrum analyzers (OSAs) to obtain the transmission spectrum. The proposed PCF is placed in a magnetic field generator to observe the transmission spectra at different magnetic field intensities. Similarly, the proposed PCF is placed in a temperature control box for measuring temperature sensitivity. The temperature and magnetic field sensing information is obtained by identifying changes in the optical spectrum.

The optical path of the magnetic fluid sensor adopts a MZI structure, and its optical path transmission is shown in Figure 3, which can generate two sets of interference. This kind of interference structure can divide the final spectrum into two states: the x-polarization state and y-polarization state. The structural analysis shows that the final interference spectrum is mainly affected by the length of the PCF and the length difference of the SMF between the sensing arm and the reference arm:

(3) $$l_{x}{=L}_{s}\times n_{\mathrm{smf}}+L_{\mathrm{pcf}}\times n_{\mathrm{eff}}{}^{x}$$

(4) $$l_{y}{=L}_{s}\times n_{\mathrm{smf}}+L_{\mathrm{pcf}}\times n_{\mathrm{eff}}{}^{y}$$

where *l*_x_ is the difference in the effective refractive index caused by x polarization, *l*_y_ is the difference in the effective refractive index caused by y polarization, L_pcf_ stands for the length of the PCF of the sensing arm, L_s_ = L_2_ + L_3_ − L_1_ represents the length difference of the SMF between the sensing arm and the reference arm, n_eff_^x^ stands for the x-polarization effective mode’s refractive index and n_eff_^y^ stands for the y-polarization effective mode’s refractive index. According to the two-beam interference theory, the output intensity, I, is as follows:

(5) $$I=I_{r}+I_{s}+2\sqrt{I_{r}I_{s}}\cos\Delta \varphi$$

where I_r_ is the light intensity of the reference arm, I_s_ is the light intensity of the sensing arm Δφ is the phase difference between the reference arm and the sensing arm, and the values in the x- and y-polarization states can be obtained via Equation (6):

(6) $$\Delta \varphi_{i}=\frac{2\pi \cdot l_{i}}{\lambda },i=x,y$$

where λ is the wavelength of light. Formula (7) can be used to calculate the interference spectrum, P:

(7) $$P=10\cdot \log\frac{1-\cos(\Delta \varphi )}{2}$$

Substituting Equations (3), (4) and (6) into (7), the final transmission spectrum can be deduced as follows:

(8) $$P_{x}=10\log\frac{1-\cos\frac{2\pi (L_{s}\cdot n_{\mathrm{smf}}+L_{\mathrm{pcf}}\cdot n_{\mathrm{eff}}{}^{x})}{\lambda }}{2}$$

(9) $$P_{y}=10\log\frac{1-\cos\frac{2\pi (L_{s}\cdot n_{\mathrm{smf}}+L_{\mathrm{pcf}}\cdot n_{\mathrm{eff}}{}^{y})}{\lambda }}{2}$$

where P_x_ is the transmission spectrum under x polarization, and P_y_ is the transmission spectrum under y polarization. If the requirement for phase matching is satisfied, there will be a peak in the interference spectrum of x polarization and y polarization. The sensitivities of the sensor to temperature and magnetic field will be determined, by monitoring the movement of the peak under different magnetic fields and temperatures.

According to the Formulas (8) and (9), the interference spectra under x and y polarization can be calculated, respectively. By substituting the correlation values for different magnetic field intensities and different temperatures in (8) and (9), the corresponding transmission spectra can be observed.

**Figure 4:**
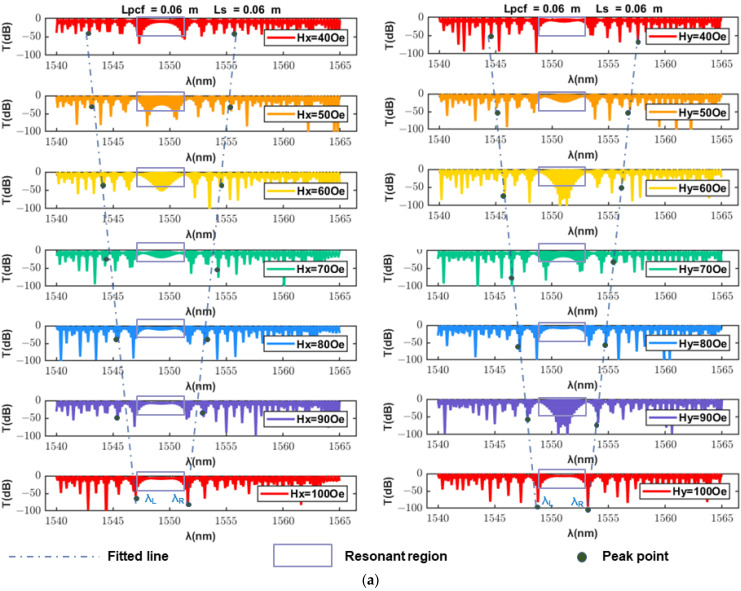

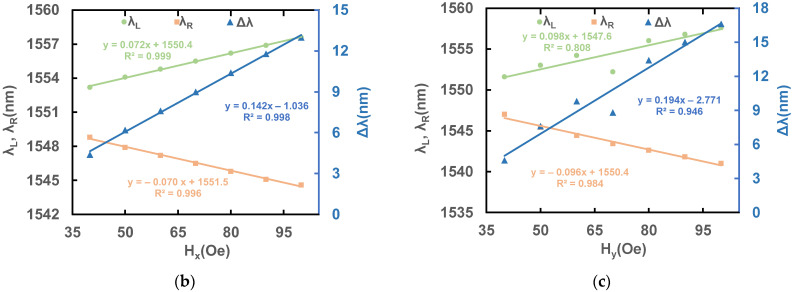
(**a**) Interference spectra under different magnetic field intensities at a wavelength of 1540–1565 nm with x and y polarization. The fitted line of λ_L_, λ_R_ and Δλ for (**b**) H_x_ and (**c**) H_y_ in the wavelength range of 1500–1600 nm.

Figure 4a shows the interference spectra under different magnetic field intensities at the wavelength of 1540–1565 nm under x polarization and y polarization. The length of the SMF is 0.06 m and the length of the petaloid PCF is 0.06 m. In this case, the temperature is kept constant at 20 °C and only the magnetic field intensity is changed. The H_x_ on the left side of the figure represents the magnetic field intensity in the x-polarized state, and the H_y_ on the right side represents the magnetic field intensity in the y-polarized state. From the top to the bottom of the figure is shown the transmission spectrum with increasing magnetic field intensity. To facilitate the observation, the spectra at different magnetic field intensities are separated. The region near 1540–1565 nm is defined as the resonant region, and the change in light intensity on both sides of it is almost symmetrical. Using the fitted line to mark the peak of the corresponding wavelength, we can see that the drift of the peak point is linear. In addition, there is a peak drift on both sides of the resonance region, and by observing the difference in the peak drift, the magnetic field sensitivity can be calculated. Similarly, the transmission spectrum can be observed by keeping the magnetic field intensity constant and changing only the temperature to calculate the temperature sensitivity.

At the wavelength range of 1540–1565 nm, the change in magnetic field intensity will affect the corresponding peak drift on both sides of the resonance region. On the right side of the resonance region, the peak wavelength on the right side decreases as the magnetic field increases, while on the left side of the resonance region, the peak wavelength on the left side regularly increases as the magnetic field increases. The change in peak wavelength at different temperatures is the same as that in the magnetic field. Then, the magnetic field and temperature sensitivity of the dual-parameter sensor can be expressed as follows:

(10) $$S_{H}=\frac{\partial (\lambda_{R}-\lambda_{L})}{\partial H}=\frac{\partial \Delta \lambda }{\partial H}(\mathrm{nm/mT})$$

(11) $$S_{T}=\frac{\partial (\lambda_{R}-\lambda_{L})}{\partial T}=\frac{\partial \Delta \lambda }{\partial T}(\mathrm{nm/{}^{\circ}C})$$

where λ_R_ is the wavelength corresponding to the peak on the right side of the resonance region, λ_L_ is the wavelength corresponding to the peak on the left side of the resonance region, S_H_ is the magnetic field sensitivity, S_T_ is the temperature sensitivity of the sensor and Δλ is the wavelength difference between the two peaks.

Figure 4b,c shows a fitted line of λ_L_, λ_R_ and Δλ for H_x_ and H_y_ in the wavelength range of 1500–1600 nm. Taking the wavelength difference between two peaks as the dependent variable, the sensitivity is nearly doubled compared with that under the single-peak wavelength method. Therefore, the wavelength difference method is chosen.

## 3. Results and Discussion

### 3.1. The Design of The Petaloid PCF

As shown in Figure 4, the corresponding λ_L,peak_ and λ_R,peak_ under x polarization and y polarization differ by only a few nm. In order to greatly increase the difference between x polarization and y polarization at the interference spectrum, we have designed the petaloid PCF structure with the high birefringence coefficient. Therefore, the influence of structural parameters on the birefringence coefficients B, *l*_x_ and *l*_y_ has been studied.

#### 3.1.1. Effect of Pore Size on PCF Performance

Firstly, the effect of aperture size on the PCF is analyzed. Since the aperture setting of the air holes near the fiber center has the greatest effect on the fiber, this paper explores the effect of the large aperture, D, of the two air holes at the left and right sides of the fiber center and the small aperture, d, of the two air holes at the top and bottom sides of the fiber center.

**Figure 5:**
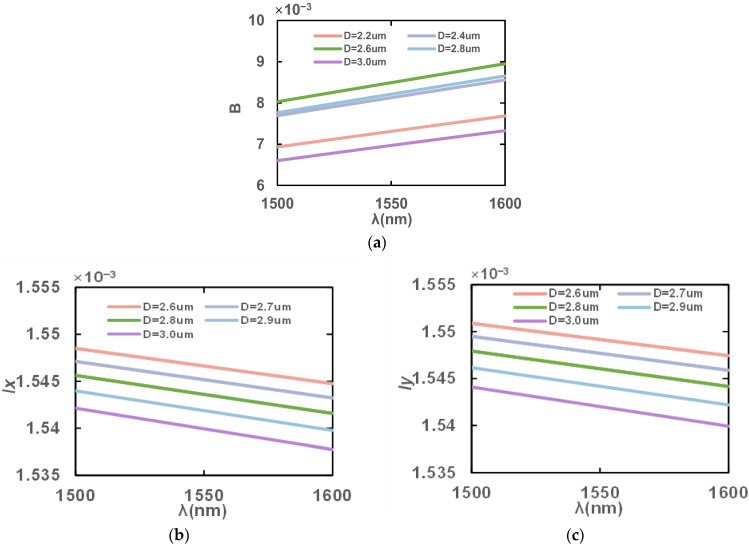
Variation in (**a**) birefringence coefficients, B, (**b**) *l*_x_ and (**c**) *l*_y_ in the wavelength range of 1500–1600 nm with different apertures, D.

Figure 5 shows the variation in birefringence coefficients, B, *l*_x_ and *l*_y_ at 1500–1600 nm for different D values with the rest of the structure being unchanged. It can be seen that as the D value increases, the birefringence coefficient, B, *l*_x_ and *l*_y_ decrease. It is because the larger aperture diameter results in the weakening of the asymmetry in the *x* direction and *y* direction, which in turn reduces the birefringence coefficients, B. In addition, a larger aperture, D, close to the core causes the proportion of pores in the fiber core to increase, so the effective mode refractive index decreases, and *l*_x_ and *l*_y_ also gradually decrease. When oriented to practical applications, the inner aperture size needs to be reasonably adjusted to increase the birefringence and maintain reasonable light field energy transmission. From the analysis, the birefringence coefficient, B, is the maximum when D = 2.6 μm, and stomatal spacing is appropriate in this case. Therefore, we choose the value D = 2.6 μm for the final design.

**Figure 6:**
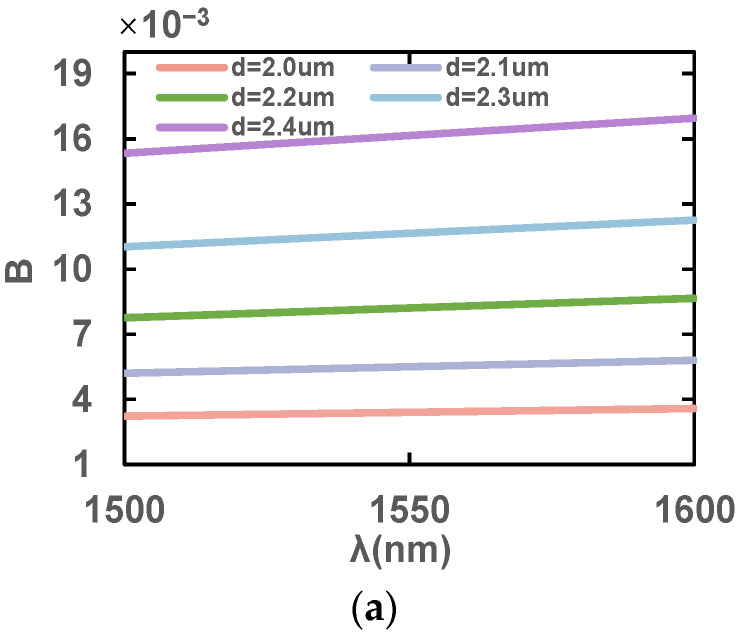

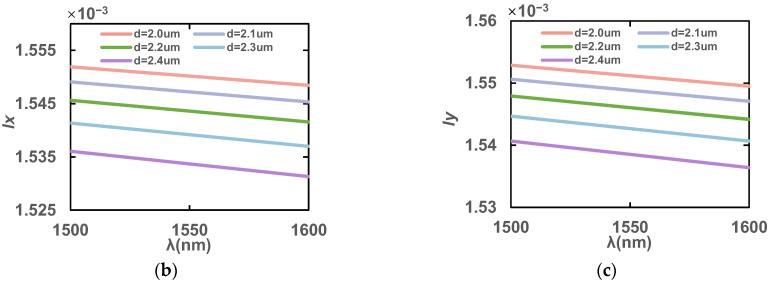
Variation in (**a**) birefringence coefficients, B, (**b**) *l*_x_ and (**c**) *l*_y_ in the wavelength range of 1500–1600 nm with different apertures, d.

Next, the effect of the small air holes on the upper and lower sides of the PCF of diameter d are analyzed. Figure 6 illustrates the variations in birefringence coefficients, B, *l*_x_ and *l*_y_ at 1500–1600 nm for different d values with the rest of the structure unchanged. It can be observed that as d decreases, B gradually decreases whereas *l*_x_ and *l*_y_ increase. This is due to the fact that a smaller d reduces the asymmetry of the fiber and thus reduces the birefringence. However, this is more favorable for the light to be bound in the core, leading to an increase in the effective mode’s refractive index and thus *l*_x_ and *l*_y_ increase as well. From the analysis, the birefringence coefficient, B, is at the maximum when d = 2.4 μm, and stomatal spacing is appropriate in this case. Therefore, the value d = 2.4 μm is finally chosen for the final design. 

#### 3.1.2. Effect of Hole Spacing on PCF Performance

**Figure 7:**
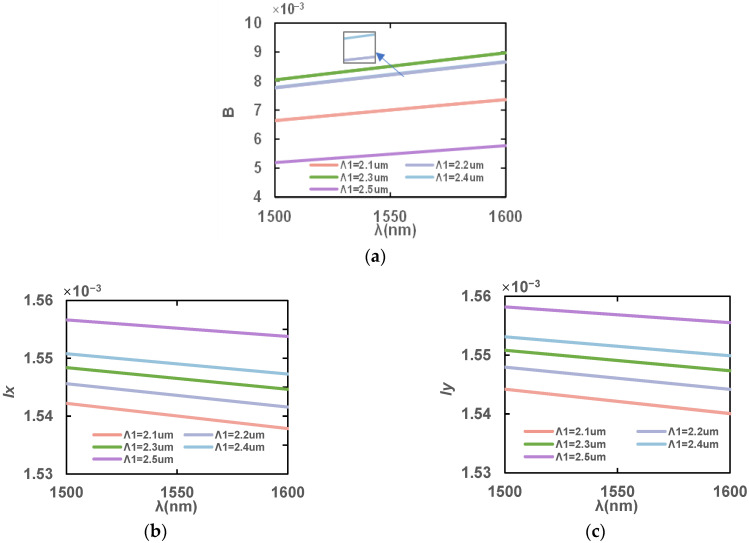
Variation in (**a**) birefringence coefficients, B, (**b**) *l*_x_ and (**c**) *l*_y_ in the wavelength range of 1500–1600 nm with different spacings, Λ_1_.

Hole spacing is critical to the design of the PCF and focuses on the innermost air hole spacing. Firstly, Figure 7 shows the effect of different Λ_1_ values on B, *l*_x_ and *l*_y_ at 1500–1600 nm with the rest of the structure parameters unchanged. The birefringence coefficient, B, increases because the larger the Λ_1_, the greater the asymmetry in the *x* direction and *y* direction. However, the later decrease in the birefringence coefficient, B, is due to the overlap between the hole’s D and the surrounding holes when Λ_1_ = 2.4 μm. With the increase in Λ_1_, the stomatal share of the core decreases and the refractive index of the effective mode increases, thus increasing the *l*_x_ and *l*_y_ of the fiber. From the analysis, the birefringence coefficient, B, is at the maximum when d = 2.3 μm, and stomatal spacing is appropriate in this case. Therefore, the value Λ_1_ = 2.3 μm is chosen as one of the final structural dimensions of the PCF. 

**Figure 8:**
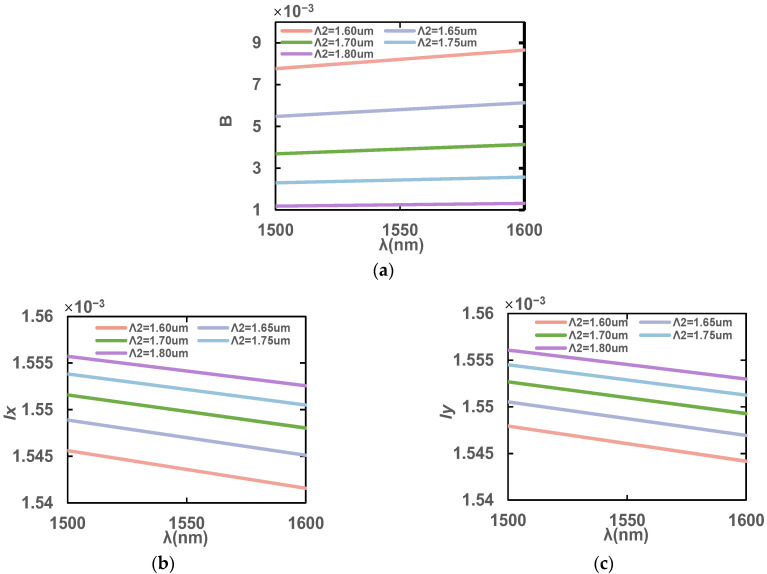
(**a**) Birefringence coefficient, B, (**b**) *l*_x_ and (**c**) *l*_y_ in the wavelength range of 1500–1600 nm with different hole spacings, Λ_2_.

Next, the influence of air hole spacing in the y direction on the PCF is analyzed. As shown in Figure 8, the influence of different Λ_2_ values on B, *l*_x_ and *l*_y_ at the wavelength of 1500–1600 nm with the rest of the structure parameters unchanged is displayed. As Λ_2_ increases, the birefringence decreases, while *l*_x_ and *l*_y_ become larger, because the larger the Λ_1_ spacing, the smaller the asymmetry in the *x* direction and the *y* direction, so the birefringence decreases. However, at the same time, the increase in Λ_2_ will also reduce the proportion of air holes in the fiber core, increasing the effective mode’s refractive index, and therefore *l*_x_ and *l*_y_ increase. Λ_2_ = 1.6 μm has the largest B, and stomatal spacing is appropriate in this case. Therefore, Λ_2_ = 1.6 μm is selected to meet the high birefringence requirement.

**Figure 9:**
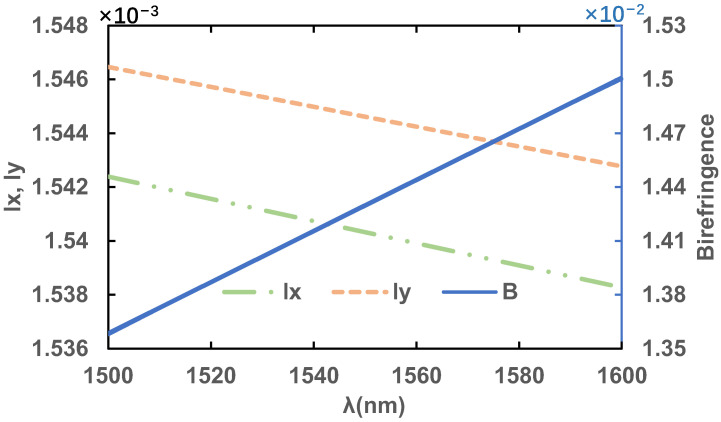
Birefringence coefficient, B, *l*_x_ and *l*_y_ in the wavelength range of 1500–1600 nm for the final design of PCF.

In summary, the final design dimensions of the PCF in this paper are D = 2.6 μm, d = 2.4 μm, Λ_1_ = 2.3 μm and Λ_2_ = 1.6 μm. Figure 9 shows the birefringence, *l*_x_ and *l*_y_ of the final designed PCF in this paper at the wavelength range of 1500–1600 nm. The birefringence coefficient with this design reaches up to 1.43 × 10^−2^ at 1550 nm, which is higher than that of the conventional PCF.

In recent years, various complex structures of PCF using the current manufacturing methods have been actually produced [32,33]. The petaloid photonic crystal fiber designed in this paper is composed of round holes with different sizes and the spacing among holes is in line with that in the actual production conditions. The fabrication method can be carried out via extrusion [34], which is relatively simple to perform and can be adapted to a wide range of complex PCF designs. The method is as follows: a mold for the desired PCF is firstly made, and the substrate material is heated to a molten state and injected into the mold. The molten material is then extruded through the mold at high temperature and pressure. This results in the production of preformed rods. In addition, Ryan et al. [35] developed a sol-gel casting technique, which has the freedom to adjust the size, shape and spacing of the holes so that PCF with any structure can be produced. Therefore, the optical fiber designed in this paper can be completely fabricated using these methods.

### 3.2. Interference Structure Design

In order to increase the measurement sensitivity to the magnetic field intensity, the magnetic fluid was injected into all the air holes of petaloid PCF for the theoretical analysis. The influence of interference structure to sensitivities of magnetic field and temperature were analyzed when the PCF was filled with magnetic fluid. It can be seen from Equations (8) and (9) that the sensitivity of the sensor is related to the length of the SMF and PCF, and the design of the sensor will be optimized accordingly.

#### 3.2.1. Magnetic Field Sensitivity Performance Design

**Figure 10:**
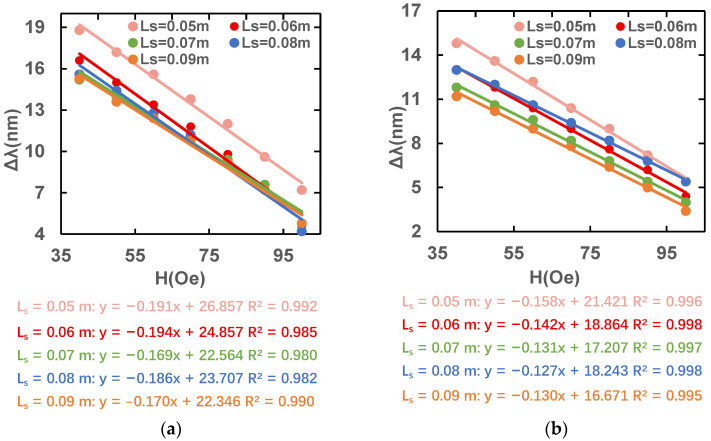
Variation in peak wavelength difference with different magnetic field intensities in (**a**) x- and (**b**) y-polarization states.

Firstly, we keep L_s_ = 0.06 m and change L_pcf_, then observe the relationship between the magnetic field intensity and peak wavelength difference. Figure 10 shows the variation of the peak wavelength difference on both sides of the resonance region when the applied magnetic field range is 40–110 Oe as determined by keeping L_s_ constant and changing only the length of the PCF. The temperature is kept at 20 °C and only the magnetic field intensity is changed. The slope of the magnetic field intensity wavelength difference is largest and the sensitivity is highest when L_pcf_ is 0.05 m under x polarization, but the slope of the magnetic field intensity wavelength difference and the sensitivity are highest under y polarization when L_pcf_ is 0.07 m. When L_pcf_ is 0.06 m, both x-and y-polarized states are at high sensitivity, so we finally choose L_pcf_ = 0.06 m as the optimal length.

**Figure 11:**
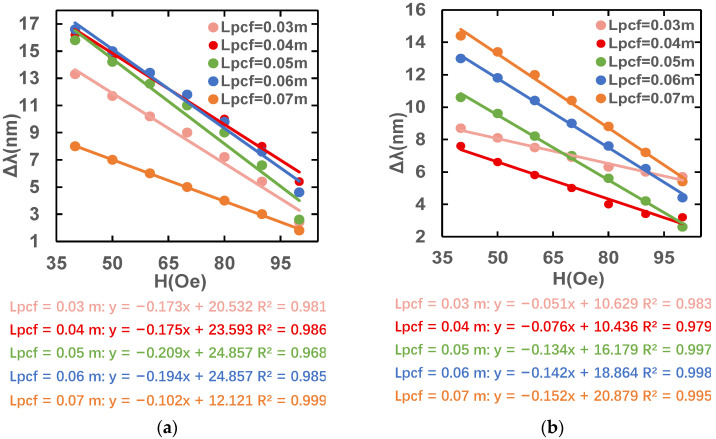
Variation in peak difference for different magnetic field intensities in (**a**) x- and (**b**) y-polarization states.

Then, we keep L_pcf_ = 0.06 m, and observe the relation between the magnetic field intensity and peak wavelength difference. Figure 11 shows the variation in the peak wavelength difference on both sides of the resonance point when the applied magnetic field range is 40–110 Oe, as determined by keeping the length of the PCF constant and changing only L_s_. The temperature is kept at 20 °C and only the magnetic field intensity is changed. The slope of the magnetic field intensity wavelength difference is largest and the sensitivity is highest when L_s_ is 0.06 m under x polarization, but the slope of the magnetic field intensity wavelength difference and the sensitivity are highest under y polarization when L_s_ is 0.05 m. When L_s_ is 0.06 m, both x-and y-polarized states are at high sensitivity, so we finally choose L_s_ = 0.06 m as the optimal length.

**Figure 12:**
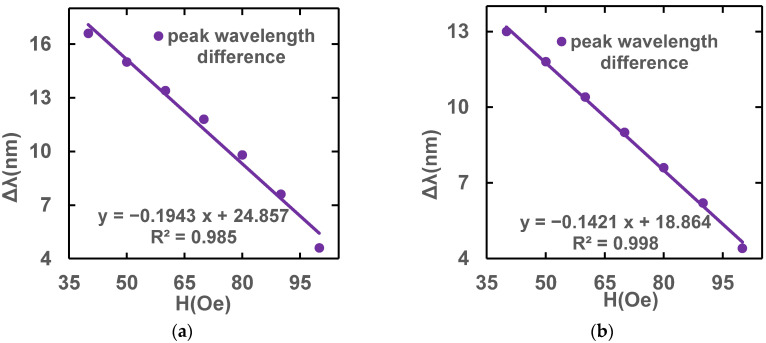
Magnetic field sensitivity fitting lines under (**a**) x-polarization and (**b**) y-polarization fundamental modes at optimal length.

In summary, the L_s_ is 0.06 m and the L_pcf_ is 0.06 m are chosen as the most suitable structure. Figure 12 shows that the magnetic field sensitivity of the sensor structure with the optimal fiber lengths in the x-polarized and y-polarized state is −0.1943 nm/Oe and −0.1421 nm/Oe, respectively.

#### 3.2.2. Temperature Sensitivity Performance Design

**Figure 13:**
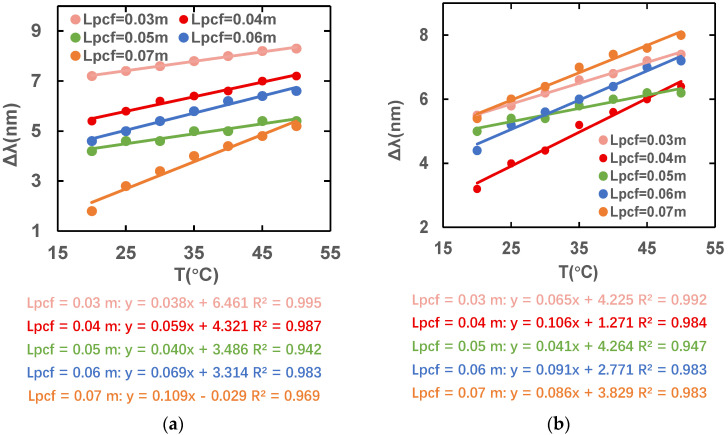
Variation in peak difference for different temperatures under (**a**) x- and (**b**) y-polarization states.

Figure 13 shows the variation in the peak difference at different wavelengths when the temperature ranges from 20 to 50 °C with the constant L_s_ = 0.06 m and different L_pcf_ values. The magnetic field intensity is kept at 40 Oe and only the temperature is changed. The slope of the magnetic field intensity’s wavelength difference is largest and the sensitivity is highest when L_pcf_ is 0.07 m under x polarization, but the slope of the magnetic field intensity’s wavelength difference and the sensitivity are highest under y polarization when L_pcf_ is 0.04 m. When L_pcf_ is 0.06 m, both x-and y-polarized states are at high sensitivity, so we finally choose L_pcf_ = 0.06 m as the optimal length.

**Figure 14:**
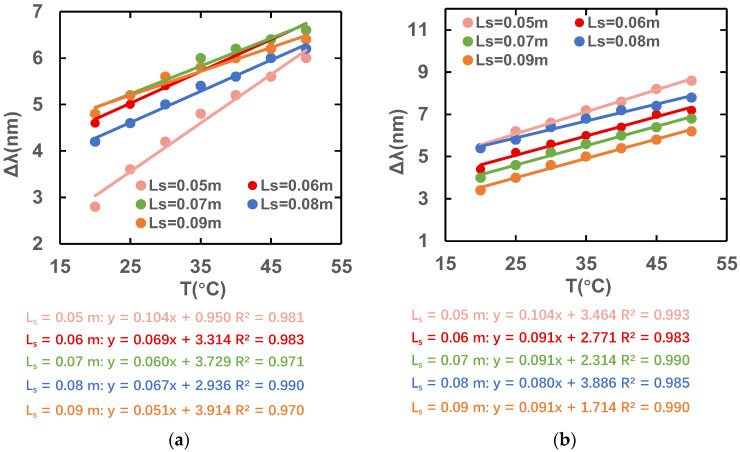
Variation in peak difference for different temperatureds in (**a**) x- and (**b**) y-polarization states.

Figure 14 shows the variation in the peak difference at different wavelengths when the temperature ranges from 20 to 50 °C with the constant L_pcf_ = 0.06 m and different L_s_ values. The magnetic field intensity is kept at 40 Oe and only the temperature is changed. It can be observed that when L_s_ is 0.05 m, the slope of the wavelength difference–temperature curve is the largest and the sensitivity is the highest. However, considering the sensitivity of magnetic field, finally, we choose L_s_ = 0.06 m as the optimal length.

**Figure 15:**
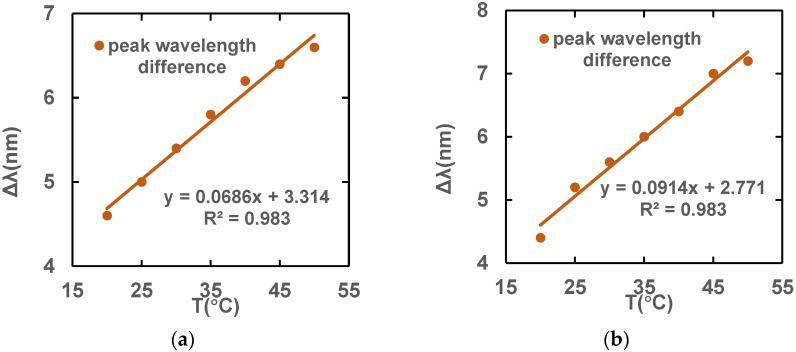
Temperature sensitivity fitting lines under (**a**) x-polarization and (**b**) y-polarization fundamental modes at optimal length.

In summary, a of Ls 0.06 m and a Lpcf of 0.06 m are chosen as the most suitable structure, coinciding with the design dimensions where magnetic field sensitivity is higher. Figure 15 shows that the temperature sensitivities for x polarization and y polarization are 0.0686 nm/°C and 0.0914 nm/°C, respectively.

Based on previous analysis results, the relation between the peak wavelength difference shift and the magnetic field intensity and temperature variation of the proposed sensor can be deduced as follows:

(12) $$\left[\begin{array}{c} \Delta \lambda^{x}\\ \Delta \lambda^{y} \end{array}\right]=\left[\begin{array}{cc} -1.943 & 0.0686\\ -1.421 & 0.0914 \end{array}\right]\left[\begin{array}{c} \Delta H\\ \Delta T \end{array}\right]$$

By simultaneously multiplying both sides of Equation (12) with the inverse matrix of the coefficient matrix, the following matrix is obtained:

(13) $$\left[\begin{array}{c} \Delta H\\ \Delta T \end{array}\right]=\left[\begin{array}{cc} -1.141 & 0.856\\ -17.738 & 24.254 \end{array}\right]\left[\begin{array}{c} \Delta \lambda^{x}\\ \Delta \lambda^{y} \end{array}\right]$$

**Figure 16:**
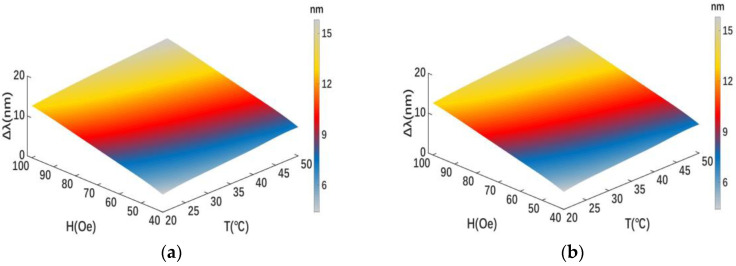
Peak wavelength difference variation with simultaneously changing magnetic field intensities and temperatures under (**a**) x- and (**b**) y-polarization states.

This matrix can be used to calculate temperature and magnetic field intensity variations as long as the peak wavelength differences of x polarization and y polarization are determined. As seen in Figure 16, when the magnetic field intensity and temperature fluctuate, the peak wavelength difference changes simultaneously.

## 4. Conclusions

This paper proposes a magnetic fluid sensor made of petaloid PCF. The petaloid PCF with a high birefringence index of 1.43 × 10^−2^ is designed by adjusting the aperture size and aperture distance of the air holes in the center of the PCF. The magnetic fluid is injected into the air holes of the designed petaloid PCF to improve the magnetic field and temperature sensitivity of the sensor. The sensor adopts a MZI structure with a simple structure and better interference effect, which can realize the dual-parameter measurement of temperature and magnetic field. The impact of the length of the PCF and the length of the SMF on temperature and magnetic field sensitivity are examined in order to improve the optical path structure. With this design, when the sensor is in the x-polarized state, the temperature sensitivity is 0.0686 nm/°C and the magnetic field intensity sensitivity is −1.943 nm/mT. When the sensor is in the y-polarized state, the temperature sensitivity is 0.0914 nm/°C and the magnetic field intensity sensitivity is −1.421 nm/mT.

The multi-parameter sensor studied in this paper provides a reliable design solution for magnetic field and temperature detection. This design has great potential and can be applied in the detection of weak magnetic fields and research on magnetic-field-sensing instruments in the future.

## Data Availability

Not applicable.
